# How do you feel during the COVID-19 pandemic? A survey using psychological and linguistic self-report measures, and machine learning to investigate mental health, subjective experience, personality, and behaviour during the COVID-19 pandemic among university students

**DOI:** 10.1186/s40359-021-00574-x

**Published:** 2021-06-02

**Authors:** Cornelia Herbert, Alia El Bolock, Slim Abdennadher

**Affiliations:** 1 Applied Emotion and Motivation Psychology, Institute of Psychology and Education, Faculty of Engineering, Computer Science and Psychology, Ulm University, Albert Einstein Allee 47, 89081 Ulm, Germany; 2 Computer Science Department, Faculty of Media Engineering and Technology, German University in Cairo - GUC, New Cairo City, Egypt

**Keywords:** COVID-19, Corona virus, Pandemic, Personality, Mental health, Depression, Anxiety, Emotion perception, Self-concept, Linguistic analysis, Machine learning, Character computing

## Abstract

**Background:**

The WHO has raised concerns about the psychological consequences of the current COVID-19 pandemic, negatively affecting health across societies, cultures and age-groups.

**Methods:**

This online survey study investigated mental health, subjective experience, and behaviour (health, learning/teaching) among university students studying in Egypt or Germany shortly after the first pandemic lockdown in May 2020. Psychological assessment included stable personality traits, self-concept and state-like psychological variables related to (a) mental health (depression, anxiety), (b) pandemic threat perception (feelings during the pandemic, perceived difficulties in describing, identifying, expressing emotions), (c) health (e.g., worries about health, bodily symptoms) and behaviour including perceived difficulties in learning. Assessment methods comprised self-report questions, standardized psychological scales, psychological questionnaires, and linguistic self-report measures. Data analysis comprised descriptive analysis of mental health, linguistic analysis of self-concept, personality and feelings, as well as correlational analysis and machine learning. N = 220 (107 women, 112 men, 1 = other) studying in Egypt or Germany provided answers to all psychological questionnaires and survey items.

**Results:**

Mean state and trait anxiety scores were significantly above the cut off scores that distinguish between high versus low anxious subjects. Depressive symptoms were reported by 51.82% of the student sample, the mean score was significantly above the screening cut off score for risk of depression. Worries about health (mental and physical health) and perceived difficulties in identifying feelings, and difficulties in learning behaviour relative to before the pandemic were also significant. No negative self-concept was found in the linguistic descriptions of the participants, whereas linguistic descriptions of feelings during the pandemic revealed a negativity bias in emotion perception. Machine learning (exploratory) predicted personality from the self-report data suggesting relations between personality and subjective experience that were not captured by descriptive or correlative data analytics alone.

**Conclusion:**

Despite small sample sizes, this multimethod survey provides important insight into mental health of university students studying in Egypt or Germany and how they perceived the first COVID-19 pandemic lockdown in May 2020. The results should be continued with larger samples to help develop psychological interventions that support university students across countries and cultures to stay psychologically resilient during the pandemic.

**Supplementary Information:**

The online version contains supplementary material available at 10.1186/s40359-021-00574-x.

## Background

Only in a few month, the COVID-19 epidemic developed into a serious pandemic affecting all countries around the globe. Physical and social distancing and global lockdown of public, social, and work life was and still is a necessity in many countries to fight the pandemic without vaccine. Scientific progress in understanding the behaviour of the virus has grown rapidly since the outbreak of the pandemic, while scientific understanding of the psychological consequences of the pandemic is still at a developing stage. Empirical studies investigating mental health, well-being, subjective experience and behaviour during the COVID-19 pandemic are currently underway and several survey studies from several countries have meanwhile been published. First published surveys investigated the mental health of Covid-19 survivors or of health care professionals enrolled in the treatment of COVID-19 patients [1, 2]. Moreover, first observations from surveys investigating psychological reactions of the general population in the hot spot countries immediately after the outbreak of the COVID-19 pandemic in 2020 have meanwhile been published e.g., [3–5]. The results suggest a significant increase in mental ill health among populations during the first few months of the COVID-19 pandemic, supporting earlier observations from previous epi- and pandemics [6]. The World Health Organization (WHO) expects mental health burdens in the general population to be particularly pronounced in people who have already been at risk of or suffering from affective disorders before the pandemic (see for an overview [7, 8]). Similarly, patients in general as well as patients with a chronic mental disorder in particular, are expected to suffer from impairments in mental health and well-being due to their medical and psychotherapeutic treatment being reduced or cancelled as a consequence of the pandemic lockdown [8]. In addition, health care professionals involved in the treatment of COVID-19 patients as well as workers with system-relevant jobs are supposed to be at special risk of developing stress-related symptoms and diseases such as post-traumatic stress disorder, chronic fatigue, anxiety, and depressive disorder [1, 2, 8].

However, the current COVID-19 pandemic is not just threatening specific parts of the population. On the contrary. The spread of the virus around the world, its exponential increase in infection probability, and its high lethality bear constant threats for whole societies and for each individual as the pandemic is still evident now, one year after the pandemic outbreak.

Therefore, according to the WHO, primary mental health prevention targeting either the general public or specific population groups should be an indispensable goal of crisis management of the current COVID-19 pandemic [8] comprising all age-groups from youth, adolescence to adulthood.

Notably, fighting the COVID-19 pandemic currently still requires behaviour change in everybody including daily behaviour (work, business, family, and leisure) as well as changes in health behaviour and social behaviour. In each country so far, the COVID-19 pandemic lockdowns affected daily behaviour routines including work, business, family, and leisure time activities. The COVID-19 pandemic lockdowns started in China in January 2020 and only a few months later, lockdowns followed in many countries around the globe including Germany and Egypt in March 2020. Crucially, in all countries, the first lockdowns came by far and large unexpected to the population. The restrictions in daily life and behaviour may therefore not be tolerated equally well by everybody. Accordingly, health care professionals and the WHO have suggested that counseling programs supporting and assisting people in behaviour change need to become part of the COVID-19 pandemic prevention initiatives [8, 9] to avoid unnecessary mental health burdens in the general public.

However, in order to successfully support mental health, well-being, and behaviour in those social domains of life most seriously affected by the current COVID-19 pandemic, a better scientific understanding is required of how individual people experience and psychologically react to the current COVID-19 pandemic, how they think, feel, suffer and cope with the situation, and how they are handling threat perception, how they perceive and regulate emotions and behaviour [10].

Academia and education are two social and public domains that have been seriously affected by the pandemic lockdown in every country. Concerning Germany, in March 2020 the different states of Germany decided to postpone all academic teaching at higher education institutions to an indefinite period. The universities’ infrastructure including libraries were closed and students were not allowed to come to the university. Similarly, concerning Egypt, public and private universities responded in a similar manner as mandated by the government by closing the campus for students and switching all teaching activities to e-learning. Teaching courses including classes, laboratory courses, seminars, preparatory and induction courses were suspended for the summer term 2020. Teaching during the summer term was announced to be offered as online e-learning format. The lockdown situation in the two countries was thus almost identical for university students concerning the aspects of their social and academic life.

Working at home without any possibility of coming to the university campus and not being able of attending to lectures and courses face-to-face together with peers, tutors, and teachers require from students to learn and adapt to new behaviour rules. Psychologically, pandemics increase uncertainty [11]. Uncertainty causes stress and increases the risk for mental ill health if it conflicts with behaviour routines and habits [11]. Despite most of the students being digital natives, the abrupt switch from face-to-face communication to digital, computer-assisted forms of teaching and sole reliance on digital interaction as the only means of social interaction might not be tolerated mentally and physically equally well by all students. Whether the current pandemic situation and its consequences are experienced as a threat may depend on the students’ individual character, i.e., the student’s personality and self-concept as well as his/her current cognitive, affective, and motivational state.

Recent observations from published survey studies among Chinese students after the lockdown reported an increase in general anxiety within about 25% of the student participants. Anxiety symptoms ranged from mild to moderate to severe anxiety [3]. Moreover, pandemic self-isolation was found to be associated with complex patterns of psychopathology amongst students including an increase in symptoms of obsessive–compulsive disorder, hypochondria, depression, and neurasthenia [4]. Meanwhile published survey studies from several countries in Europe and across the world support negative changes in mental health among university students immediately after the first lockdowns in 2020, specifically in relation with quarantine and self-isolation [12–16].

Nationwide surveys conducted before the COVID-19 pandemic already reported elevated mental health problems and stress-related symptoms including anxiety and depression among university students [17–21], and this, although university students across countries might belong to the young educated low-risk population. In a recent online study including N = 185 university students studying in Germany, 36.6% of the students (women and men) reported to experience depressive symptoms, 41.83% (women and men) reported high levels of state anxiety, and mental stress due to excessive demands and uncertainty in finances, job, or social relationships [21]. This prevalence of academic stress and mental health burdens have been found among university students all over the globe [17–20], including Egypt [22, 23].

Thus, as a population group, university students may be particularly vulnerable to stress-related lifestyle changes affecting mental health that are associated with the current COVID-19 pandemic. Individual differences in mental health may also exist and influence how the students perceive and how well they adapt and cope with the current COVID-19 pandemic situation and to what degree they are motivated to change their behaviour in response to the pandemic consequences in social and academic life and teaching. Psychological theories and models of behaviour change, e.g., Health Belief Model, Transtheoretical Model, or Social Cognitive Theory [24–26], all agree in that individual factors, specifically those related to emotion- and self-regulation can explain how people perceive themselves, whether and why they change their behaviour and why others do not. Threat perception has been suggested to play an outstanding role [27], because pandemics threaten the whole person, i.e. our self and the self-concept. Personality traits although considered stable may play a critical role in threat perception, in mental health and behaviour because they influence and modulate the person’s feelings, beliefs, and the person’s trust in one’s own self-regulatory abilities required to change one’s own behaviour [27]. Moreover, stable personality traits and a positive self-concept are considered general important stress buffers and protectors of mental health, whereas neuroticisms, trait anxiety, difficulties in describing and identifying feelings as well as an overall negative self-concept are considered significant risk factors of mental ill-health, specifically of anxiety disorder and depressive disorder [28–30].

These examples underscore the complexity and dynamics of how individual traits and state-like individual psychological factors as well as characteristics of the situation interact and influence subjective experience and behaviour. Methodologically, this raises questions of how interactions between situation, person and behaviour can best be assessed, investigated, modeled and predicted in relation to the COVID-19 pandemic in which little empirical evidence is available so far and different aggregated data measures of qualitative and quantitative origin might be used to best capture the internal personal variables of interest (e.g., feelings, worries, self-concept, or personality traits) that provide insight into the subjective experience and the perceived changes in health and behaviour of individual persons behaving in the context of the COVID-19 pandemic.

**Fig. 1:**
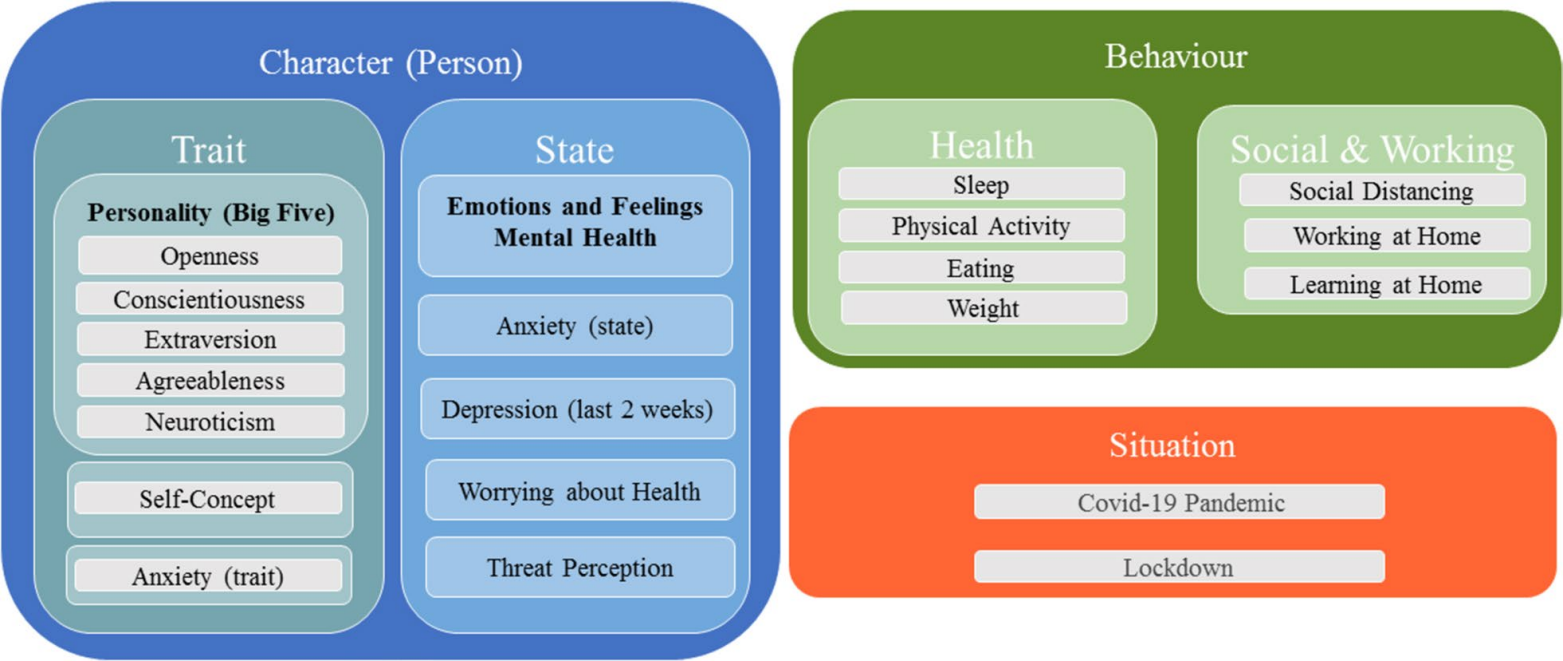
Illustration of the dynamic relationships between situation, a person’s character (traits and states), and behaviour change

Computational modeling and machine learning have been already successfully applied in the field of pandemic research to predict transmission rates of the virus based on global behavioural changes of the general population [31]. These approaches require huge data sets (big data). In health behaviour research, first attempts have been made to apply computational models to data sets comprising smaller sample sizes to model behaviour of individuals, for instance, in response to behavioural interventions supporting health prevention [32]. These computational models build on psychological theories of human behaviour. Character Computing is one of these psychologically-driven approaches, whose computational models include stable character traits (e.g., personality, self-concept) and cognitive, affective, and motivational state variables and behavioural indicators as input to take into consideration the dynamic interactions between situation (S), person (P) and behaviour (B) (for an overview, see [33–35] and Fig. 1). The computational models are not fixed but can be improved and extended, e.g., by ontologies [36] or automated data processing, the more empirical evidence and data is available [32–35].

## Methods

### Aim of this online survey study

Based on the challenges of the COVID-19 pandemic outlined above, this online survey study is aimed at contributing to the scientific understanding of the psychological consequences of the pandemic by investigating mental health, subjective experience, and behaviour among university students studying in Egypt or Germany after the first pandemic lockdown in May 2020. As outlined above, university students may be particularly sensitive to lifestyle changes related to the COVID-19 pandemic, negatively affecting the students’ mental health, their subjective experience and behaviour. Moreover, as also explained above, the students’ personality traits and self-concept might constitute important stable psychological variables that could influence mental health as well as subjective experience and behaviour related to the COVID-19 pandemic. Therefore, to fully capture these psychological aspects, psychological assessment included a number of psychological variables ranging from stable personality traits and self-concept to state-like psychological variables sensitive to situational change and related to (a) mental health (current depressive symptoms and state anxiety), (b) pandemic threat and emotion perception including current feelings, (c) worries about health including perceived changes in paying attention to bodily symptoms, and (d) self-reported perceived changes in health behaviour (weight, eating, sleeping, physical activity), social and learning behaviour (difficulties in self-regulated learning). To capture all aspects summarized under (a)–(d), the assessment methods comprised a mix of self-report tools (survey items, standardized psychometric scales, psychological questionnaires, and linguistic self-report measures).

Data analysis included (a) descriptive analysis for prevalence estimation of mental health variables, (b) linguistic analysis of self-concept, personality and feelings during the pandemic and (c) correlational analysis and machine learning tools. Machine learning tools were used for exploratory purpose only to further explore the idea of whether machine learning algorithms could despite small sample sizes be trained to predict stable personality traits from the self-report data of the students. Knowing whether stable personality traits (that due to their stability cannot easily be changed by health care interventions) can be predicted from the students’ self-report data could help develop individualized health care interventions that take the students’ personality development into account. The online survey was distributed among university students studying at universities in Egypt and also in Germany. Both countries were equally affected by the lockdowns in May 2020. With respect to the already published survey studies (see above), all attesting an increase in mental ill health among university students during the COVID-19 pandemic the following main research questions were addressed:

- *RQ1* Mental health: Can the present online survey study confirm high state anxiety and depressive symptoms reported in previous studies in the current sample of university students during the time period of the first COVID-19 pandemic lockdown in May 2020? Crucially, are the self-reported symptoms of anxiety and depression when assessed on standardized psychological screening and assessments tools beyond the cut off scores of clinical samples, and comparable or even higher than the prevalence rates reported in pre-pandemic surveys?
- *RQ2* Threat perception and worries about health: Do university students report to experience threat, negative feelings and worries about health during the COVID-19 pandemic?
- *RQ3* Emotion perception: Do university students report to perceive difficulties in emotion perception in the time period of the first pandemic lockdown relative to before the pandemic?
- *RQ4* Health behaviour, social behaviour and learning: Do university students report to perceive changes in health behaviour (e.g., weight, eating, sleeping, physical activity, paying attention to bodily symptoms), and do they report to experience difficulties in self-regulation during learning (teaching), and in social behaviour in the time period of the first pandemic lockdown?
- *RQ5* Self-concept and personality: Do university students report a positive or a negative self-concept? Are mental health variables correlated with the students’ personality?
- *RQ6* Exploratory analysis: Can machine learning despite small data sample sizes predict stable personality traits from the self-report data of the students?

### Participants

The survey study was designed and conducted by the Department of Applied Emotion and Motivation Psychology of Ulm University and administered via Ulm University and LimeSurvey software (https://www.limesurvey.org/de/). The survey was advertised among others via the university’s international office to reach specifically students studying in Egypt. The survey was provided in English language (i.e., the academic language), and proficiency in English language was a prerequisite for taking part in the study. Participants were fully debriefed about the purpose of the survey, participation was voluntary and anonymous (see ethics statement). After registration, participants answered questions about their language proficiency, age, gender, their university, study year, and their living situation (alone, with friends or family). Only university students who were aged 18 years and older, and who provided informed consent were able to participate in the study. The survey items were structured in blocks of items and questionnaires: sociodemographic (1), personality (Big-Five) and anxiety (state and trait) (2), survey items about teaching, survey items about health including the linguistic task (self-concept) (3–4), and finally, emotion perception and depression screening (5). The blocking of the serial order of these topics lead to partial drop-outs across the survey, particularly across blocks (see below).

**Fig. 2:**
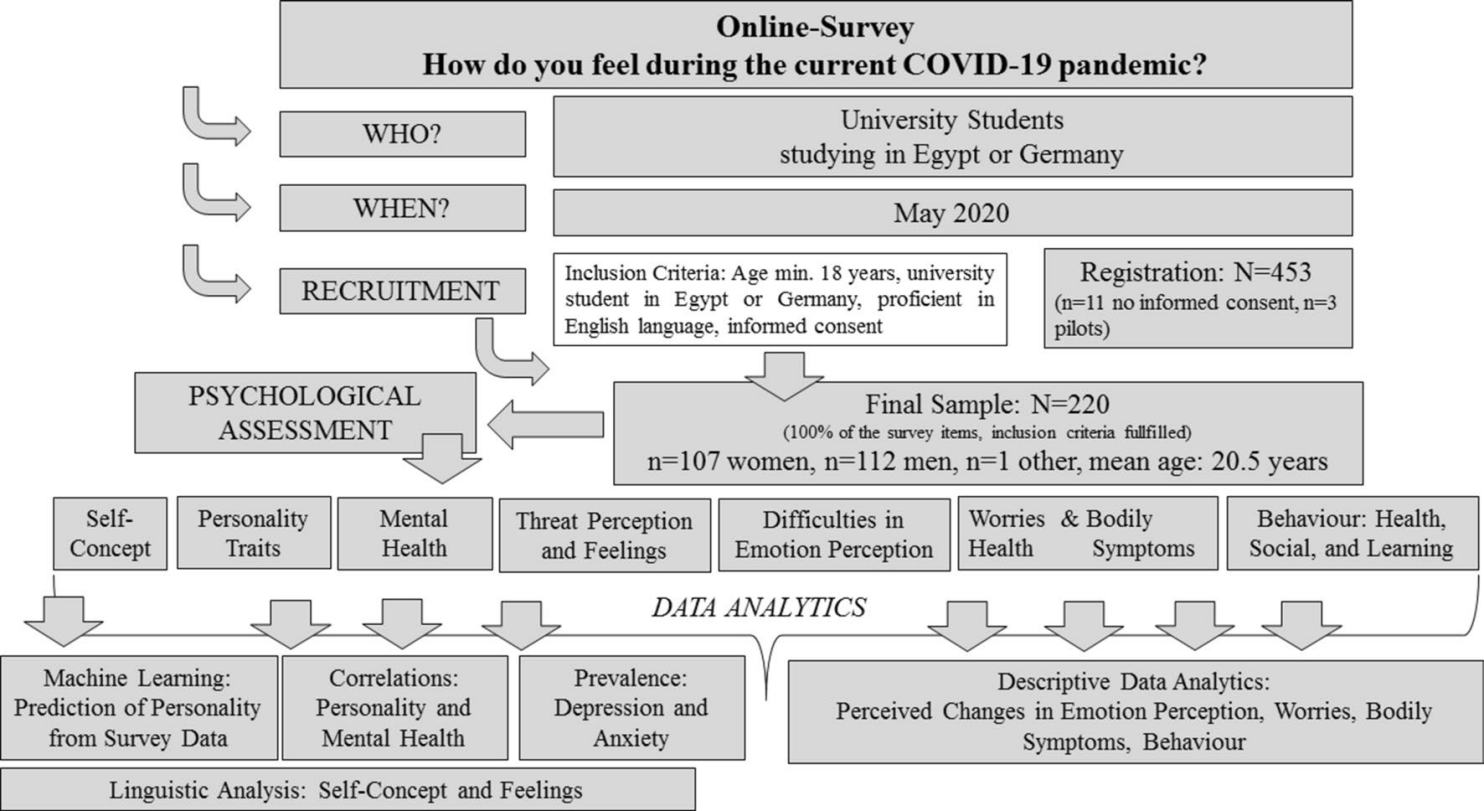
Design of the survey including data collection and recruitment of participants and data analytics. Please see sections ““Aim of this online survey study” and “Methods” for detailed explanation

An overview of the complete study-design is provided in the flow-diagram in Fig. 2. An overview of the online survey items and questionnaires can be found in the Additional file 1.

### Study sample, survey drop-out and missing data

In total, N = 453 university students registered for the study and answered the inclusion and exclusion criteria. Of these, n = 3 were pilots and n = 11 participants did not give informed consent or did not explicitly state that they want to get their data published in scientific research, and were therefore excluded from the study sample. N = 439 volunteers (n = 215 men, n = 219 women, n = 5 did prefer not to name their gender; *mean age*: 20.69 years, *SD* = 2.87 years) completed the sociodemographic questions. Of these, n = 19 (4.3%) did not report to study in Germany or Egypt and were excluded. Of the 420 university students who reported to study in Egypt or Germany, n = 325 participants (n = 167 men, n = 156 women, n = 2 did prefer not to name their gender; *mean age*: 20.38 years, *SD* = 1.76 years, range: 18–33 years) filled in the personality and anxiety questionnaires only, while n = 220 participants (n = 112 men, n = 107 women, n = 1 did prefer not to name the gender; *mean age*: 20.45 years, *SD* = 1.88 years, range: 18–33 years) completed the entire survey. This corresponds to a survey completion rate of 0.49 (division of the number of participants who complete the entire survey (n = 220) by the total number of participants who register for the survey (n = 453)). This rate falls within the rate expected for online surveys (20–50%).

**Fig. 3:**
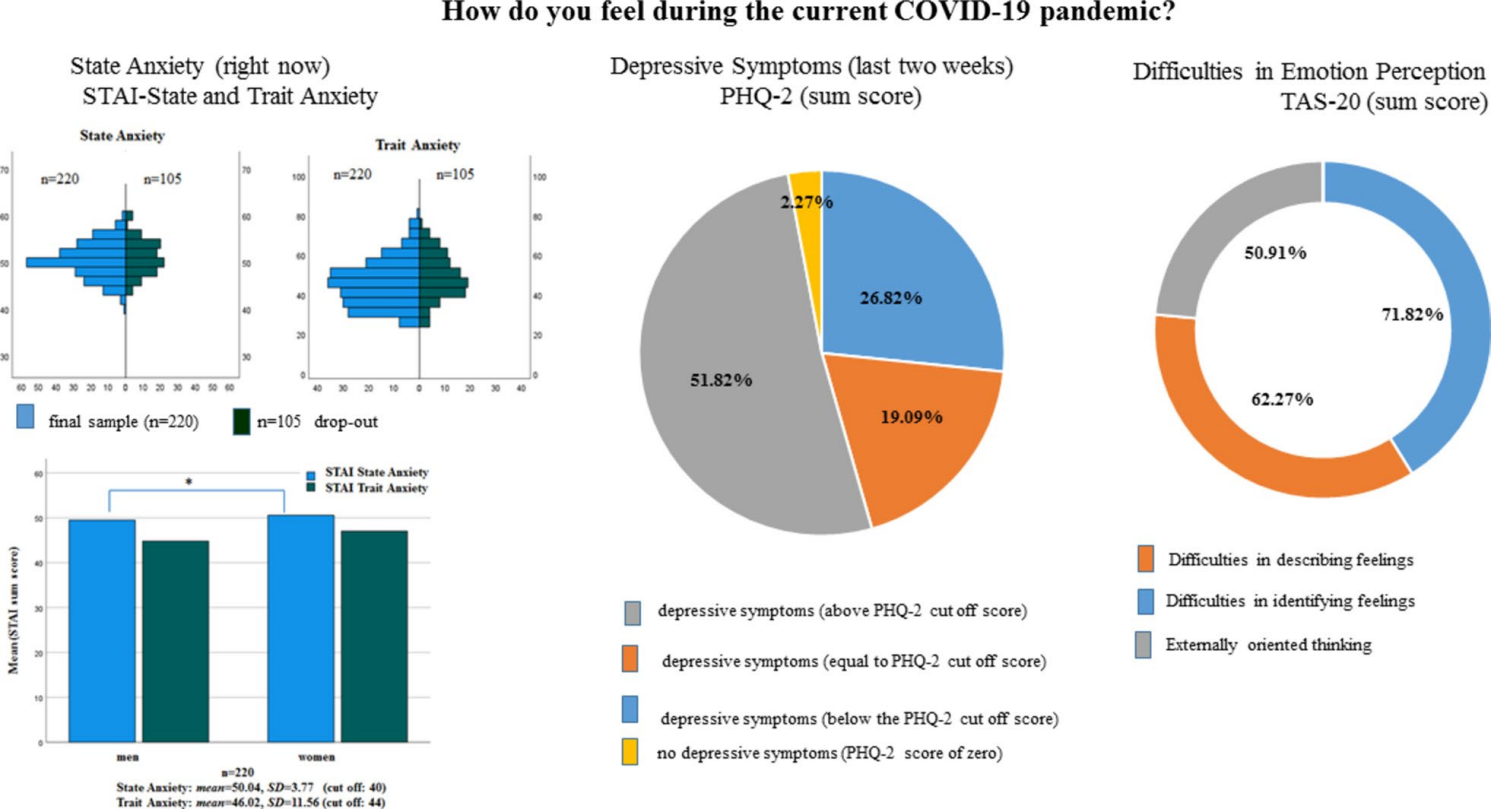
State and Trait Anxiety distributions across the final sample and drop outs (left upper column). Mean state and trait anxiety scores in women and man in the final sample (left lower column), significant results (*p* < .05) are illustrated by lines and cross. Percentage of students reporting depressive symptoms (middle column). Right column: Percentage of students reporting changes in emotion perception on the TAS-20 questionnaire and subscales after the COVID-19 pandemic outbreak

Analysis of the drop-outs (including e.g., univariate measures of variance (ANOVA)), showed no difference in age between the groups (i.e., the sample who filled in the sociodemographic items only (n = 95) versus the sample who filled in the personality and anxiety questionnaires only (n = 105) versus the final sample (n = 220), *F*(417,2) = 1.72, *p* = .18. In addition, the student samples did not differ with respect to gender, i.e., the % of the number of women and men. Analysis of anxiety and personality scores likewise suggests that the final sample and the sample who dropped-out after filling in the personality or anxiety questionnaires (n = 220 versus n = 105) did not differ in state anxiety or in the scores on any of the Big-Five personality dimension. (state anxiety: *F*(323,1) = 1.77, *p *> .18; Openness: *F*(323,1) = 0.16, *p *> .69; Conscientiousness: *F*(323,1) = 2.82, *p *> .13; Extraversion: *F*(332,1) = 0.94, *p *> .33; Agreeableness: *F*(323,1) = .062, *p *> .43; Neuroticism: *F*(323,1) = 1.22, *p *> .27). Mean scores of trait anxiety differed between the final sample and the sample who dropped out (n = 220: *mean*: 46.02, *SD* = 11.2, *range*: 26–79 vs. n = 105: *mean*: 49.02, *SD* = 10.98, *range*: 26–77, *F*(323,1) = 5.78, *p* = .017). However, using median tests (which are less susceptible to outliers) showed no significant difference in the distribution of trait anxiety scores between the samples (median-test = 1.59, *p* = .21), see Fig. 3 for an overview.

The survey was programmed such that it produced as little missing data as possible. Therefore, missing data of single items in a questionnaire or in a block of open items could be excluded and missing scores were therefore not imputed. Regarding the self-generated prompts, participants were free to answer the prompts (self-concept and feeling descriptions). Inspection of the data shows that in the full sample, 5 participants did not fill in all of self-descriptive prompts, leaving open 1, 2 or 3 of the descriptions, respectively.

### Measures: survey items and questionnaires

The online survey included several self-report measures comprising a mix of single items with open and closed questions, standardized psychometric scales, and standardized psychological questionnaires. The section below and Table 1 provide an overview of the survey items, questionnaire measures and hypotheses grouped according to the psychological domains and research questions of interest (for an overview, see also RQ1–RQ6 in the section “Aim of this online survey study”).

**Table 1:** Overview of the self-report measures included in the survey and hypotheses

Questionnaires and survey items	Hypotheses
**Mental Health**	
**Depression**	
*Questionnaire*: PHQ-2 [38]	
Screening for depressive symptoms Cut off score (> 3): risk of depression Symptom assessment (last 2 weeks)	- Presence of depressive symptoms - Prevalence of depressive symptoms in the present student sample probably higher than reported in surveys before the pandemic
**Anxiety**	
*Questionnaire*: STAI [37]	
STAI-State (how do you feel right now) STAI-Trait (anxiety proneness, how do you feel in general) Cut off scores: (> 40/44) high versus low state anxiety, high versus low trait anxiety	- High prevalence of state anxiety and trait anxiety, probably higher than reported in surveys before the pandemic
**Threat perception**	
*Survey Items*: How does the current pandemic situation make you feel?	
• Answers on 9-point SAM scales [41] (valence, arousal, dominance)	
(a) valence (negative/unpleasant-positive/pleasant, 1–9) (b) arousal (low/calm-high/aroused; 1–9) (c) dominance (low/no control–high/in control; 1–9)	- Higher negativity/unpleasantness than positivity/pleasantness - High arousal ratings - Lack of dominance (not in control of the situation)
• Answers: discrete emotions:	
- happy, neutral, surprise, disgust, anger, fear, or sadness Scales: “yes”, “no” (“no” indicates no change)	- Feeling more often afraid, angry, sad than happy or surprised or neutral
**Feelings during the pandemic**	
*Survey Item*: Describe your feelings in response to the COVID-19 pandemic by completing the prompt “I feel ….” Answer: free text, five words	High number of negative words expressing negative feelings than positive words expressing positive feelings
**Difficulties in emotion perception** *(relative to before the pandemic)*	
*Questionnaire*: TAS-20 [42]	
Cut off score > 60	
3 subscales: - difficulties on describing feelings - difficulties in identifying feelings - externally oriented thinking	- Difficulties describing and identify feelings and externally oriented thinking style
**Worries about health and perceived changes in health behaviour during the pandemic**	
*Single survey items* (created for this survey)	
- worries about mental health - worries about physical health - perceived changes in physical activity ○ exercise less (one item) ○ exercise more (one item)	- Worries in mental and physical health expected - Perceived changes expected in all health domains (less physical activity, more eating, weight gain, and change in sleeping)
- perceived changes in eating behaviour ○ eat more (one item) ○ eat less (one item) - perceived changes in sleeping behaviour ○ sleep more (one item) ○ sleep less (one item)	
- perceived changes in weight ○ weight gain (one item) ○ weight loss (one item)	- Increase expected in all bodily domains
Answers: now during the pandemic relative to before the pandemic Scales: Scales: “yes”, “no” (“no” indicates no change) - perceived changes in paying attention to bodily sensations and symptoms ○ taste ○ smell ○ cardiovascular ○ breathing/respiration ○ appetite/eating/drinking Answers: now during the pandemic relative to before the pandemic Scales: Likert type (1 = not at all/decreased, 10 = increased/very much)	
**Social behaviour**	
*Single survey items* (created for this survey)	
Following pandemic rules (social distancing) Difficulties in not going out Answers: now during the pandemic relative to before the pandemic Scales: Scales: “yes”, “no” (“no” indicates no change)	- Difficulties in social distancing
**Teaching and Learning behaviour**	
*Single survey items* (created for this survey) Difficulties in self-regulated learning - Attention and effort: ○ unable to concentrate and focus ○ preoccupation with the current situation, lost in content Answers: now during the pandemic relative to before the pandemic Scales: Scales: “yes”, “no” (“no” indicates no change)	- Difficulties expected in self-regulatory capacities
**Personality and Self-Concept** *BIG Five* *Questionnaire*: BFI-40 [43]	
Subscales - neuroticism - extraversion - openness - conscientiousness - agreeableness *Self-Concept* *Linguistic task* modified according to TST [45]	- Personality traits are expected to be correlated with self-reported changes in anxiety, depression and emotion perception
Describe your personality “I am …” Answer: free text, five words	- Positive versus negative self-concept should be associated with positive or negative word use, respectively

For detailed description, please also see sections “Methods”, and “Measures” and “Hypotheses”

### Mental health: anxiety (trait/state), current depressive symptoms (last 2 weeks)

As illustrated in Table 1, the participants anxiety proneness including trait and state anxiety as well as their current self-reported depressive symptoms (last 2 weeks) were assessed with psychological questionnaires including the Spielberger Trait and State Inventory (STAI, [37]), and the Patient Health Questionnaire (PHQ-2, [38]). The STAI is available in many different languages and has shown similar values of internal consistencies among university students from European and Arabic countries [39]. Whereas the trait scale of the STAI asks for how one generally feels, the instruction of the state scale of the STAI asks for how one feels right now. The PHQ-2 has proven to be a robust screening for depressive symptoms across different cultures including European and Arabic countries [40]. It asks for the presence of depressive symptoms over a time period of the last two weeks.

### Threat perception, feelings, and perceived difficulties in emotion perception during the COVID-19 pandemic

Threat perception as well as discrete emotions and feelings in response to the COVID-19 pandemic situation were assessed by single survey items. Specifically, these items asked the participants about how the current COVID-19 pandemic situation makes them feel in terms of valence (positive/pleasant-negative/unpleasant), arousal (low/calm-high/aroused), and dominance (feeling in or out of control of the situation). The 9-point Self-Assessment Manikin scales (SAM, [41]) were used for valence, arousal and dominance assessment. The SAM scales are one of the most robust and frequently used scales for the unbiased, non-verbal assessment of emotions and feelings on the three dimensions of emotions including valence, arousal and dominance [41]. In accordance with the literature [41], the SAM scales ranged from 1 (negative/unpleasant, low arousal/calm, out of control) to 9 (positive/pleasant, high arousal/aroused, in control). In addition, we asked the participants to indicate which kind of discrete emotions they experienced in response to the COVID-19 pandemic. Participants could choose among six discrete emotions (sad, anxious, angry, disgusted, happy, surprised, or neutral). In addition, participants were given five prompts to describe their current feelings in response to the COVID-19 pandemic situation (“I feel ….”). In order to assess potential difficulties in emotion perception, participants filled in the Toronto Alexithymia Scale (TAS-20; [42]), which comprises the three subscales “Difficulty Describing Feelings”, “Difficulty Identifying Feelings”, and “Externally-Oriented Thinking”. Since we were interested in perceived changes since the pandemic outbreak, participants were instructed to answer each item of the TAS-20 questionnaire relative to before the pandemic.

### Worries about health and perceived changes in behaviour during the COVID-19 pandemic

Worries about health, perceived changes in paying attention to bodily symptoms (e.g., taste, smell, cardiovascular, respiration/breathing, appetite/eating/drinking), as well as perceived changes in health behaviour (weight, eating behaviour, sleep and physical activity behaviour) as well as perceived difficulties in social behaviour (social distancing) and self-regulatory learning (i.e., difficulties in paying attention to the content provided by e-learning, difficulties in studying with the same effort as before the pandemic situation) were assessed via single survey items. The single item questions that asked for worries and perceived changes in behaviour could be answered with “yes” or “no”; “yes” meaning an increase and “no” meaning no change in relation to before the pandemic. The items on health behaviour included items asking in both directions, e.g., whether one eats more or less, sleeps more or less, exercises more or less than before the pandemic. The single item questions of paying attention to bodily symptoms could be answered on 10-point Likert scales such that change scores could be calculated based on the participants’ answers allowing evaluation of the degree of change as increase, decrease or no change during the pandemic situation in relation to before the pandemic (see Table 1 for an overview).

### Personality and self-concept

As illustrated in Table 1, the participants’ personality traits were assessed with the Big Five Personality Inventory (BFI-40, [43]). The BFI-40 is a standardized self-report measure that has been validated in different cultural populations and age groups [44]. The self-concept was assessed using a modified short version of the twenty statements tests (TST, [45]). The TST is a cross-cultural tool for the assessment of different facets of the self-concept including actual, ideal, and ought selves. In the present study, participants had to generate self-descriptions for the actual self only. In line with the instruction of the TST [45], participants were asked to provide five words to the prompts “I am ….” in order to describe themselves.

### Hypotheses

#### Mental health: anxiety (trait and state) and current depressive symptoms

In line with previous pre-pandemic surveys among university students (see Background for an overview), we expected a high prevalence of anxiety and depressive symptoms in the present sample of university students irrespective of their culture or country in which they study. Prevalence rates for self-reported current depressive symptoms assessed with the screening tool of the PHQ-2 asking for depressive symptoms in the last 2 weeks (PHQ-2 items: item1: “little interest or pleasure in doing things”; item 2: “feeling down, depressed or hopeless”) and state anxiety (asking for how one feels right now) might be expected to be even higher than prevalence rates reported in previous surveys before the pandemic situation.

#### Threat perception, feelings, and difficulties in emotion perception

We expected threat perception to the COVID-19 pandemic to be associated with self-reported unpleasantness, feelings of moderate to high levels of arousal, self-reported perceived lack of dominance (feeling less in control of the situation) on the Self-Assessment Manikin (SAM) scales. In addition, we expected self-reports of feelings of anger, sadness, and anxiety towards the pandemic as assessed by the survey items assessing discrete emotions. We also explored whether students report to perceive changes in emotion perception since the pandemic outbreak relative to before the pandemic outbreak. Specifically, we explored whether participants report difficulties in describing and identifying feelings and report externally oriented thinking on the TAS-20 as potential maladaptive adaptions in coping with the pandemic lockdown. As mentioned above, the instruction of the TAS-20 items asked the participants to answer the items in relation to before the pandemic.

#### Worries about health, perceived changes in behaviour during the COVID-19 pandemic

We expected that the majority of students will report to be more worried about their mental and physical health than before the pandemic. Moreover, we expected a higher awareness of bodily symptoms (i.e., paying more attention to perceived changes in smell, taste, cardiovascular functions, breathing/respiration, and appetite/eating/drinking) relative to before the pandemic. Given that the lockdown in every country had effects on the students’ work and leisure time activities, we also expected that participants will report changes in health behaviour including a decrease in regular physical activity compared to before the pandemic lockdown including self-reported changes in eating- and sleeping behaviour and weight. We also expected difficulties in learning and social behaviour (see Table 1).

#### Personality and self-concept

Moreover, we examined how university students see themselves (self-concept). In particular, we explored whether the students would report a positive or negative self-concept and compared their linguistic descriptions of the self to their descriptions of their current feelings pandemic-related feelings (“I feel …) and their personality. Regarding personality, we explored whether stable psychological personality traits (Big Five and trait anxiety) would be correlated with state anxiety and depressive symptoms and the students’ perceived changes in emotion perception. Finally, we examined for exploratory purpose, whether machine learning could predict the students’ personality traits from their reports (for details see “Data Analysis” section).

#### Descriptive analyses and statistics

To answer the hypotheses outlined above, the participants’ answers (questionnaires, single items) were analysed descriptively to provide insight into how many students on average reported anxiety and depressive symptoms as well as how many students reported to perceive changes in subjective experience (threat perception, difficulties in emotion perception, worries about health, bodily symptoms) and behaviour (health, social, learning). Analysis of the questionnaires (PHQ-2, STAI, TAS-20, BFI-40) followed the guidelines and manuals and were calculated as sum scores or mean scores (non-normalized). For the PHQ-2, STAI and TAS-20, cut off scores are available from the literature (see “Results” section). These cut off scores were also used in the present study to discriminate between high versus low trait anxiety, high versus low state anxiety, depressive symptoms, and difficulties in emotion perception. Means and standard deviations were calculated for all questionnaire data and for the closed survey items using Likert scales or the SAM scales. The questionnaire data and answers to the survey items were tested statistically for significance by means of non-parametric or parametric statistical tests as appropriate. The respective test statistics are presented in brackets in the “Results” sections. Given the drop-out across blocks of the survey (see section about Sample size, survey drop-out and missing data), the results for each scale, item or questionnaire were calculated for the available sample who filled in the questions and the final sample (n = 220) who filled in the complete survey and who reported to study in Egypt or Germany. *P* values are reported uncorrected and two tailed if not otherwise specified. The SPSS software (IBM SPSS Statistics Software, Version 27) was used for all statistical testing including correlation analysis (see below).

#### Correlational analysis

Correlation analyses (Pearson) were used to assess the relationships between the Big Five personality traits (BFI-40), mental health variables (STAI: trait and state anxiety, PHQ2: screening for depressive symptoms), and difficulties in emotion perception (TAS-20). *P* values are reported uncorrected and two tailed if not otherwise specified.

#### Linguistic analysis of self-concept and feelings

The open-ended linguistic answers assessing the self-concept (“I am …”) and feelings in response to the pandemic (“I feel …”) were analysed with computer-assisted text analysis tools including Linguistic Inquiry of Word Count (LIWC; [46]). The dictionary of the LIWC software contains words and word stems, grouped into semantic categories related to psychological constructs. The categories provided by the LIWC allow the assessment of the polarity of words (positive or negative). The LIWC analysis produces reliably results with about 500 words and more. Therefore, in the present study, words generated by each participant were accumulated across participants and entered as a whole text corpus for words generated for the prompts “I am …” (self-concept) or for the prompt “I feel …” (feelings in response to the pandemic), respectively. This allows the evaluation of the self-concept and current pandemic feelings of the university sample as a whole. For the linguistic analysis no statistic testing was performed.

#### Machine learning (exploratory analysis)

Machine learning (ML) was used for exploratory purpose only and the ML algorithms were chosen to combine the different psychological variables that were descriptively analysed in order to explore whether individual personality traits including the Big Five and trait anxiety can be predicted and classified by automated machine learning tools. To this end, the questionnaire scores and answers to the different survey items were preprocessed according to the following procedure: the participants’ Big Five personality traits from the BFI-40, the state and trait anxiety scores (from the STAI including for each individual, a difference score for self-reported trait and state anxiety), depression (PHQ-2), perceived changes regarding difficulties in emotion perception (TAS-20) as well as the participants’ answers on the SAM scales for threat perception (e.g., valence, arousal, dominance) were normalized (z-scores). The participants’ answers to the discrete emotions elicited during the pandemic, difference scores assessing increase in current anxiety (difference score comparing STAI state vs. STAI trait) as well as the participants’ answers to the survey items asking for worries and perceived changes in health and behaviour were labeled as positive or negative or set to zero if the students reported no change. The answers to the survey items asking for perceived changes in paying attention to bodily sensations/symptoms were combined to a total score denoting the total perceived changes in attention towards bodily sensations/symptoms and the total change was labeled as positive or negative depending on whether attention increased or decreased relative to before the pandemic or set to zero if there was no change. Sociodemographic variables such as country or university were no contribution factors in prediction and classification. After data preprocessing and data labeling, the dataset for machine learning comprised continuous features and discrete categorical features. The whole dataset was denoted “X” and the continuous or discrete features were denoted “y” in the feature matrix. The machine learning libraries of the Python software package (https://www.python.org/) were used for automated data analysis. Data analysis was based on regression models. Gradient Boosting Regression (GBR) and Support Vector Regression (SVR) were chosen for the regression models. The principle of Gradient Boosting Regression is to build multiple regression models based on decision trees. Decision tree models are supervised machine learning algorithms that have tree structures that recursively break down the dataset into smaller datasets through branching operations while comparing the final node results with the target values. Decision tree models provide the best fit for small sample sizes to avoid overfitting the data. The same holds true for support vector machine algorithms. Support Vector Regressions (SVR) aim at finding the best fitting line in continuous data within a predefined threshold error. The evaluation of the accuracy of the prediction is evaluated based on the root mean squared error (RMSE). Depending on the type of data to be predicted, RMSE within 10–20% of the range is considered a good result. Especially with human self-report, data accuracies are usually much lower than in other more deterministic domains of machine learning e.g., natural language processing or bioinformatics. One reason for the lower accuracies in human behaviour data is the higher variance in the data itself [47]. To account for this, we accepted a RMSE of up to 16.6% as sufficient for the decision that the data can be predicted by the model accurately.

We used the classical train/test split approach with a ratio of 8:2. Train/test split is a common validation approach frequently used in ML studies including those with smaller sample sizes [for a critical review see [48]). No k-fold cross validation (CV) approach was chosen as it has been shown that k-fold CV can lead to overestimation especially with small sample sizes, whereas train/test split and nested CV approaches have been shown to be equally reliable even with small sample sizes [48]. We also performed hyperparameter tuning, an algorithm frequently used and recommended in machine learning to choose and select during training the best model while avoiding biasing the data, and the number of features and the feature-to-sample ratio) was kept in an optimal range (less features than samples) for avoiding overfitting [48].

## Results

### Descriptive data analytics

#### Mental health: anxiety (trait and state) and depressive symptoms

The mean state and trait anxiety scores of the university students who completed the entire survey and who studied in Egypt or in Germany (n = 220) were above the cut off scores that according to the literature distinguishes between high versus low anxious subjects [49]. The mean state anxiety score as measured with the STAI inventory was significantly above the cut of score of 40 (n = 220, *mean*: 50.04, *SD* = 3.77; *T* = 39.47, *df* = 219, cut off: 40, *p* < 0.001). A cut off score below or above a score of 44 in the trait STAI scale differentiates between low trait anxious and high anxiety prone individuals [49]. The mean score for trait anxiety was significantly higher than this cut off score (n = 220, *mean:* 46.02, *SD* = 11.56; *T* = 2.60, *df* = 219, cut off: 44, *p* < 0.01). Given the drop-out of n = 105 students, the analysis of the mean state and trait anxiety scores were recalculated for the final sample including those students who dropped out. The analysis showed that also in this larger sample of n = 325 students the cut off scores were significantly above the cut off scores (state anxiety: n = 325; *mean*: 50.23, *SD* = 3.75; *T* = 49.13, *df* = 324, cut off: 40, *p* < 0.001; trait anxiety: n = 325; *mean*: 47.08, *SD* = 11.52; *T* = 4.72, *df* = 324, cut off: 44, *p* < 0.001) and in addition, trait anxiety scores (trait) did not differ significantly between women and men in this sample (trait anxiety: n = 325; *mean-woman*: 47.94, *SD* = 11.82; men: 45.96, *SD* = 10.91; *F*(321,1) = 2.45, *p* > 0.12). However, women reported higher state anxiety scores than men. This difference in state anxiety scores between women and men was significant (state anxiety: n = 325; *mean-woman*: 50.81, *SD* = 3.62; men: 49.63, *SD* = 3.79; *F*(321,1) = 8.08, *p* < 0.005) and was also significant in the n = 220 sample. There was no significant difference in state anxiety scores between students studying in Egypt or Germany, neither in the n = 220 sample nor in the sample comprising n = 325 students (n = 220, state anxiety: Egypt-*mean*: 50.16, *SD* = 3.75, Germany-*mean*: 49.08, *SD* = 3.86, *Mann*–*Whitney-U* = -1.39, *p* = 0.16; n = 325, state anxiety: Egypt-*mean* = 50.32, *SD* = 3.70, Germany-*mean*: 49.45, *SD* = 4.22, *Mann*–*Whitney-U* = -1.24, *p* = 0.22). However, students studying in Egypt reported higher trait anxiety compared to the students studying in Germany (n = 325, trait anxiety: Egypt-*mean*: 47.62, *SD* = 11.60, Germany-*mean*: 42.24, *SD* = 9.75, n = 220, trait anxiety: Egypt-*mean*: 46.49, *SD* = 11.57, Germany-*mean*: 42.40, *SD* = 10.93), but this difference was not significant in the final sample (n = 220, *Mann*–*Whitney-U* = − 1.39, *p* = 0.16). The results are illustrated and summarized in Fig. 3.

For the PHQ-2 screening for depressive symptoms a sum score greater than 3 on both items is associated with depression proneness [38]. In the sample of university students who completed the entire survey and therefore had filled in the PHQ-2 depression screening, the mean sum score was *mean:* 3.48, *SD* = 1.58, and significantly above the cut off score (*T* = 4.51, *df* = 219, cut off = 3, *p* < 0.0001). 51.82% (n = 114) of the students had sum scores greater than the cut off (> 3), and 19.09% (n = 42) had a sum score of 3 (cut off). Only 26.82% (n = 59) of the sample scored below the PHQ-2 cut off score (< 3), and only 2.27% (n = 5) did report to not suffer from loss of interest or pleasure in doing things (PHQ-2 item 1) or from feeling down, depressed or hopeless during the last two weeks (PHQ-2 item 1) (see Fig. 3 for an overview on state anxiety and depressive symptoms). The PHQ-2 scores did not differ between students studying in Egypt or Germany (n = 220, Egypt-*mean*: 3.51, *SD* = 1.56, Germany-*mean*: 3.24, *SD* = 1.79, *Mann*–*Whitney-U* = − 0.643, *p* = 0.52) nor did they differ between women and men (n = 220, woman-*mean*: 3.48, *SD* = 1.54, men-*mean*: 3.47, *SD* = 1.63, *F*(217,1) = 0.00, *p* = 0.98).

#### Threat perception, feelings, and difficulties in emotion perception

Descriptive analysis of the items assessing threat perception (SAM; Self-Assessment Manikin scales ranging from 1 (unpleasant, not aroused, or no control) to 9 (pleasant, very highly aroused, in control)) showed that, the students (n = 220) felt slightly unpleasant (*mean*: 4.19, *SD* = 1.97). In addition, 55% (n = 120) of the final study sample (n = 220) reported a score from 1 to 4, i.e., from high unpleasantness to moderate unpleasantness on the 9-point SAM valence scale. On average, the students did not feel much in or out of control of the situation (*mean*: 5.07, *SD* = 2.41) on the 9-point SAM scale for dominance. Nevertheless, 37.55% of the study sample reported a score from 1 (no control) to 4 (loss of control) on the SAM scale for dominance. Mean physiological arousal was rated as moderate (*mean*: 5.40, *SD* = 2.22). However, 50% of the university students (n = 110) reported an arousal score of 6 (aroused) to 9 (very high arousal) on the SAM arousal scale. Given the drop-out of students, comparisons of the ratings (valence, arousal, or control) were performed between samples (n = 220 and n = 59 who completed the ratings but did not fill in the entire survey). This showed that the ratings did not differ between the samples (*Mann*–*Whitney-U*-tests, all *p* > 0.70). From the set of discrete emotions (including sadness, anger, fear, disgust, happiness, surprise, or neutral emotions), 66.8% reported to feel not neutral, 93.2% reported to feel not happy, 56.4% reported to feel sad, 75.9% reported to feel angry, 92.3% reported to feel surprised, 87.7% reported to feel disgusted, and 52.7% reported to feel afraid by the current pandemic situation. The distribution of “yes” versus “no” answers differed significantly for the categories feel neutral, happy, surprised, disgusted, or angry, respectively, (non-parametric test for binomial distribution: all *p* < 0.001). From all students who completed these items (n = 277) the same significant results were obtained for the answers concerning discrete emotions.

16.88% of the students of the final sample (n = 220) had a total TAS-20 score greater than the critical TAS-20 cut off score (TAS-20 cut off > 60, [30]). From the three subscales of the TAS-20 questionnaire, changes in self-reported difficulties in emotion perception in relation to the pandemic as compared to before the pandemic were reported by 62.27% (n = 137) for items belonging to the subscale “Difficulty describing feelings”, and by 71.82% (n = 158) for the items belonging to the subscale “Difficulty identifying feelings” and by 50.91% (n = 112) for the items belonging to the subscale “Externally Orienting Thinking”. The distributions of the TAS-20 scores of the three subscales did not differ between students studying in Egypt or Germany (*Mann*–*Whitney-U*, all *p* > 0.50). However, woman (n = 107) reported higher scores on the subscales “Difficulties identifying feeling” compared to men (n = 112), *F*(217,1) = 217.1, *p* = 0.035.

#### Worries about health

**Fig. 4:**
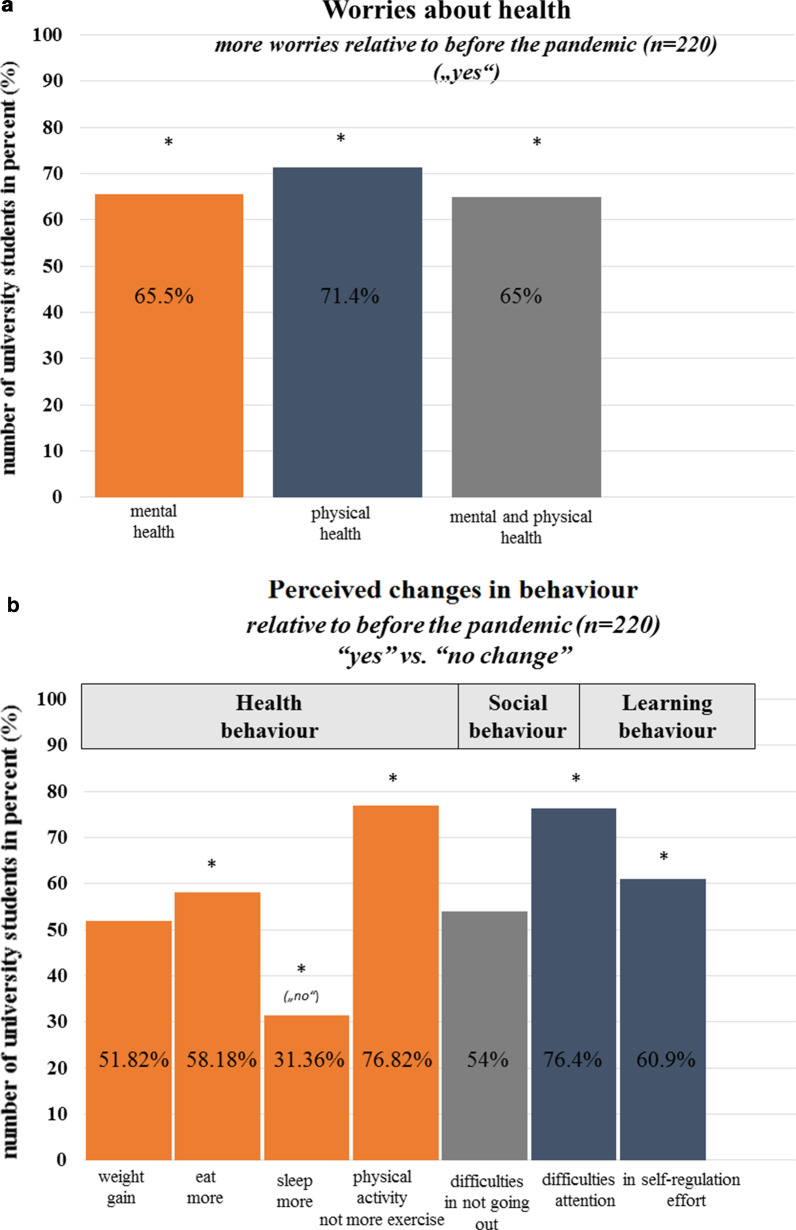
**a** Worries about mental health or physical health or both (mental and physical health). The cross represents significant results, *p* < .05. **b** Perceived changes in health behaviour including weight, eating, sleeping, and physical activity. The cross represents significant results, *p* < .05

In the final sample who completed the survey (n = 220), 65.5% (n = 144 students) of the study sample reported to worry about their mental health more due to the COVID-19 pandemic than before the pandemic, whereas 34.5% (n = 76) answered to worry not more than before the pandemic. 71.4% (n = 157) of the students reported to worry more about their physical health than before the pandemic, whereas 28.6% (n = 63) answered to worry not more about their physical health than before the pandemic. The distributions of “yes” versus “no” differed significantly for both, worries about mental and physical health, respectively (non-parametric test for binomial distribution: all *p* < 0.001) and this also held true when considering all students who filled in these items (n = 227). Self-reported worries about mental health and physical health were significantly related (χ2 = 100.43, *df* = 2, p < 0.001). 65% (n = 143 of n = 220) reported to worry in both domains (mental health and physical health) more than before the pandemic and this also held true when considering all students who filled in these items (n = 227), see Fig. 4a.

### Behaviour: health

Across health behaviour domains (weight, eating, sleep, physical activity), 52.3%, 58.2%, 31.8%, and 76.4% of the study sample (n = 220) reported to have gained weight, to eat more than before the pandemic and to not sleep more or exercise more than before the pandemic situation. The distributions of “yes” versus “no” answers were significantly different for the domains of eating, sleep and exercise/physical activity (non-parametric test for binomial distribution: eat, sleep, exercise/physical activity all *p* < 0.001) and this again held true when considering all students who filled in the items (n = 227). Paying attention to bodily sensations and symptoms (i.e., changes in taste, smell, appetite/eating/drinking, cardiovascular functions, breathing/respiration) did however not change significantly relative to before the pandemic outbreak. On average, on Likert scales ranging from 1 (“decrease”) to 5 (“no change”) to 10 (“increase”), participants reported not to pay more attention to or to be more aware of bodily sensations and symptoms than before the pandemic (smell: *mean*: 5.18, *SD* = 1.21, taste: *mean*: 5.15, *SD* = 1.27, bodily symptoms: *mean*: 5.84, *SD* = 1.74, cardiac symptoms: *mean*: 5.78, *SD* = 1.66, breathing: *mean*: 5.77, *SD* = 1.64, eating and drinking/appetite: *mean*: 5.52, *SD* = 2.09). The answers on these rating scales did not differ between students studying in Egypt or Germany (all *p* > 0.16), but comparisons between women and men showed that women scored significantly higher on the scale asking for attention to bodily symptoms than men (woman-*mean*: 6.18, *SD* = 1.90, men-*mean*: 5.50, *SD* = 1.53, *F*(217,1) = 8.50, *p* > 0.002). This again held true when considering all students who filled in the items (n = 227).

### Behaviour: social distancing and learning

Being asked about their social situation of self-isolation, teaching and learning behaviour, 54% of the student sample (n = 220) replied to have difficulties in not going out during the pandemic. 76.4% replied to have difficulties in self-regulated learning, being unable of focusing their attention on the teaching content. Of these students, 60.9% replied to have difficulties in studying with the same self-regulatory effort because of being anxiously preoccupied with the current pandemic situation (see Fig. 4b). The distributions of “yes” versus “no” answers were significantly different for the domains of learning (non-parametric test for binomial distribution: eat, sleep, exercise/physical activity all *p* < 0.002) and this again held true when considering all students who filled in these items (n = 305, all *p* < 0.001).

### Linguistic self-concept and self-descriptions of current feelings

**Fig. 5:**
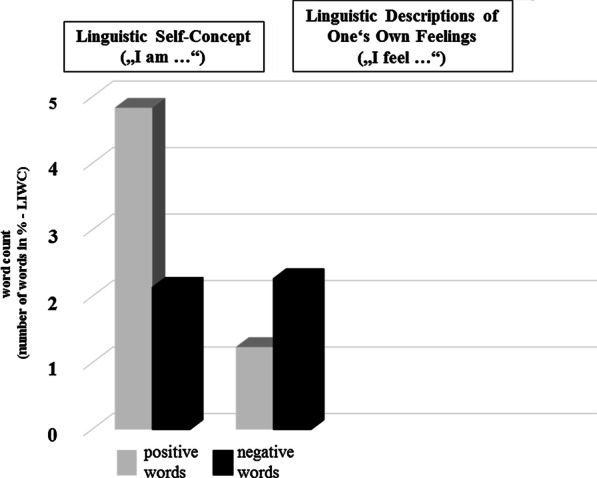
Percentage of negative and positive words. Left column: Self-concept: “I am …”. Right column: Current feelings during the pandemic “I feel …”

**Fig. 6:**
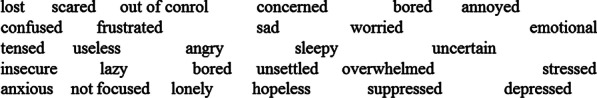
Summary of the words most often used by the university students to describe their feelings in response to the pandemic

Linguistic self-descriptions (“I am …”) showed a positivity bias. Overall, more positive words than negative words were used by the students to describe themselves (see Fig. 5). As mentioned above, linguistic analysis of the university students’ self-descriptions about how the current COVID-19 pandemic situation makes them feel (“I feel …”) showed the reverse pattern with more negative words than positive words being used by the study sample to complete the prompt “I feel ….” (see Fig. 5). In addition, Fig. 6 shows the most prominent examples, i.e., the words most often used by the students to describe their feelings during the pandemic.in the prompt “I feel …”.

### Personality: Big Five

The final student sample (n = 220) scored low on the BFI-40 subscales for extraversion (*mean*: 24.5, *SD* = 5.65), neuroticism (*mean*: 25.37, *SD* = 6.51), and reported moderate scores on the conscientiousness scale (*mean*: 30.69, *SD* = 6.07), the openness scale (*mean*: 36.85, *SD* = 5.07), and the agreeableness scale (*mean*: 33.42, *SD* = 4.50) and as described earlier (see section “Study sample, survey drop-out and missing data”), the BFI-40 scores of the samples (n = 220 vs. n = 105 who dropped-out) did not differ in the five personality dimensions. The Big Five personality traits were significantly correlated with self-reported depressive and anxiety symptoms as well as with the self-reported difficulties in emotion perception. Table 2 shows a summary of the correlations between measures of personality traits (BFI-40), trait anxiety (STAI-trait scale), state anxiety (STAI-state scale), self-reported depressive symptoms (PHQ-2), and perceived difficulties in emotion perception (TAS-20) as obtained from the final sample (n = 220).

**Table 2:** Correlations (r) between personality (Big Five), depression, anxiety and perceived difficulties in emotion perception during the COVID-19 pandemic (*p* is reported two-tailed)

Personality Big Five (BFI-40)		
	Depression	Difficulties in emotion perception
	(PHQ-2)	(TAS-20)
Conscientiousness	r = − 0.25, *p* < .001	
Neuroticism	r = 0.41, *p* < .001	r = 0.39, *p* < .001
	Anxiety	Anxiety
	(STAI-state)	(STAI-trait)
Conscientiousness		r = − 0.29, *p* < .001
Extraversion		r = 0.31, *p* < .001
Neuroticism	r = 0.39, *p* < .001	r = 0.61, *p* < .001

Mental health		
	Anxiety	Difficulties in emotion perception
	(STAI-State)	(TAS-20)
Depression (PHQ-2)	r = 0.24, *p* < .001	r = 0.54, *p* < 0.001
	Depression	
	(PHQ-2)	
Anxiety (STAI-Trait)	r = 0.37, *p* < .001	r = 0.36, *p* < .001

### Automated data analytics, machine learning (exploratory)

The university students’ personality traits (Big Five) and trait anxiety could be predicted from the psychological variables (trait and state) summarized in Table 3 through feature importance extraction by Support Vector Regression. The table and the numbers in percent show the major contributing factors to the prediction of the respective trait listed in the left column (under “Measure”). Table 4 shows the prediction accuracy suggesting that prediction of all trait attributes have similar error rates.

**Table 3:** Prediction of personality traits based on the psychological variables (trait and state) assessed in this survey by means of machine learning (ML) algorithms

Measure	x-Features and % of importance for classification of the y-feature						
STAI-trait	18.86%	17.03%	13.99%	8.14%	6.46%	5.99%	5.41%
	Extraversion	Neuroticism	STAI-State	Conscientiousness	Openness	Threat perception discrete emotions: neutral	Difficulties in social behaviour
Openness	28.68%	10.97%	9.28%	7.27%	6.51%	6.10%	
	Difficulties in emotion perception: identifying feelings	Worry about mental health	Conscientiousness	Agreeableness	Difficulties in social behaviour	Neuroticism	
Conscientiousness	20.85%	15.57%	6.94%	5.49%	5.08%	5.05%	
	STAI-Trait	Openness	Depressive symptoms	Attention to bodily symptoms	Difficulties in emotion perception: describing feelings	Extraversion	
Extraversion	28.74%	15.71%	8.57%	7.00%	5.11%		
	STAI-trait	Openness	Difficulties in social behaviour	Conscientiousness	Paying attention to bodily symptoms		
Agreeableness	18.10%	8.48%	7.65%	7.13%	6.15%	4.73%	
	Threat perception Perceived changes in valence (negative/unpleasant/positive/pleasant)	Neuroticism	Paying attention to bodily symptoms	Threat perception: Discrete emotions: feeling sad	Age	Conscientiousness	
Neuroticism	33.23%	21.94%	12.55%	8.25%	7.05%	4.64%	
	Threat perception: current anxiety (STAI state vs trait)	STAI-State	STAI-Trait	Difficulties in emotion perception: describing feelings	Perceived changes in eating behaviour	Difficulties in social behaviour

**Table 4:** Prediction accuracies of personality traits (Big Five) and trait anxiety

Feature	RMSE	% of RMSE in range
Neuroticism	0.8074366162	13.45727694
Conscientiousness	0.7932855771	13.22142629
Agreeableness	0.8710934236	14.51822373
Extraversion	0.8959132559	14.9318876
STAI-trait	0.9024231185	15.04038531
Openness	0.9794451559	16.32408593

## Discussion

The COVID-19 pandemic is taking its toll. Concerns have been raised by the WHO (2020) [8], that the COVID-19 pandemic will cause “a considerable degree of fear, worry and concern in the population” (cited from WHO, 2020 [8]) and that stress and anxiety as well as depression will increase considerably during the COVID-19 pandemic, rendering affective disorders a public mental health concern of the COVID-19 pandemic [8]. In the present survey, mental health (depressive symptoms, state and trait anxiety), subjective experience (threat perception, current feelings, perceived difficulties in emotion perception, worries about health during the pandemic) as well as perceived changes in behaviour (related to health, social behaviour and learning/teaching) was assessed among university students studying in Egypt or Germany, respectively. The survey was administered in May 2020, shortly after the lockdown in these countries. Going beyond previous surveys, the students’ self-concept and the Big Five of human personality were additionally assessed to explore psychological patterns between personality traits, mental health, and perceived changes in subjective experience by means of correlation analysis and machine learning.

### Mental health among university students

Regarding pandemic risk groups, previous cross-cultural pre-pandemic surveys have shown high prevalence rates of anxiety and depression among university students across countries [17–22, 50–53]. Therefore, the WHO’s concerns about the psychological consequences of the COVID-19 pandemic on mental health and well-being might affect university students as a population group as well. The results obtained from this sample of university students who study in Egypt or Germany during the first lockdown period confirm these concerns. In particular, the results confirm previous pre-pandemic results about mental health of university students and they seem to confirm the concerns of the WHO regarding mental health and threat perception during the current pandemic. The mean state anxiety score (assessed with standardized questionnaires including the Spielberger Trait-State Anxiety Inventory, STAI) was significantly above the cut off score that, according to the literature [34], discriminate high from low anxious subjects. In addition, state anxiety scores were significantly higher in woman than man. Moreover, 51.82% (n = 114) of the students had sum scores greater than the cut off (> 3), and 19.09% (n = 42) had a sum score of 3 (cut off). Only 26.82% (n = 59) of the sample scored below the PHQ-2 cut off score (< 3), and only 2.27% (n = 5) did report to not suffer from loss of interest or pleasure in doing things (PHQ-2 item 1) or from feeling down, depressed or hopeless during the last two weeks (PHQ-2 item 1), and self-reported depressive symptom did not differ among students studying in Egypt or Germany or in woman or men (see Fig. 3 for an overview on state anxiety and depressive symptoms). Thus, in total, 51.82% and 19.09% of the final student sample (n = 220) reported depressive symptoms at and above the cut off score for depressive symptoms [38], thus feeling depressed or hopeless and reporting a loss of interest and pleasure in the items of the PHQ-2 questionnaire during most of the days of the last 2 weeks of the COVID-19 pandemic. Prevalence rates from previous surveys among university students reported a prevalence of anxiety symptoms or depressive symptoms above 35% among university students before the pandemic (e.g., for depression or anxiety [17–22, 50–53]). A recent online study [21], including N = 185 university students studying in Germany found that 36.6% of the university students (women and men) report experiencing depressive symptoms, 41.83% (women and men) reported experiencing high levels of state anxiety, and all students reported experiencing stress due to excessive demands and uncertainty in finances, job, or social relationships. These prevalence rates have actually been found in cohort studies including university students all over the globe, irrespective of culture before the outbreak of the pandemic [17–22, 50–53]. In relation to these pre-pandemic prevalence rates, the prevalence of state anxiety and of depressive symptoms in the current sample seem to have more than doubled during the pandemic time period.

The scores for state anxiety need to be seen in relation to the results obtained for trait anxiety. As mentioned above, trait anxiety scores were even higher in those students who dropped-out, however state anxiety scores did not differ across students who completed the survey and those who did not. Students with high state anxiety during the pandemic may be at special risk of suffering from anxiety proneness in the long run. Therefore, surveys among university students should be continued to further explore the development of anxiety and particularly also of depressive symptoms during the current pandemic as well as the comorbidity of anxiety with depressive symptoms as a consequence of the COVID-19 pandemic. Very recent surveys among university students from Greece (Europe) and the United States conducted in a similar time period (during the first lockdowns in these countries) report similar high percentage numbers of anxiety, depression and mental health burdens [12, 13]). Given that the STAI asks for feelings of stress, worry, discomfort, experienced on a day to day basis one could expect changes in other psychological domains as well (see below).

### Threat perception and perceived difficulties in emotion perception

Being asked about their feelings during the pandemic, 55% of the students reported unpleasantness and 37.55% of the students rated to be in loss of control of the situation, and about 50% reported moderate to high physiological arousal. Moreover, university students reported a mix of discrete emotions in response to the pandemic. In particular, there was a significant loss of happiness, and a change in feelings of surprise, disgust and anger. In line with this, as illustrated in Fig. 5, linguistic analysis of the participants’ answers to the questions “I feel …” also suggest a negativity bias in the linguistic descriptions of the students’ feelings: In summary, there was more intense use of negative than positive words to describe one’s feelings in response to the pandemic. Thus, feelings of threat and negative emotions were also reflected in the self-generated linguistic answers of the students, supporting a general increase in anxiety during the first period of the COVID-19 pandemic among university students. Similarly, and in line with the scores obtained from the depression screening instrument (PHQ-2), linguistic analysis of the questions “I feel …” revealed a high percentage of words such as feeling depressed, down or hopeless (see Fig. 6). Thus, anxiety and depression related words were amongst the most frequently used words when participants were asked to describe in their own words, how the current COVID-19 pandemic situation makes them feel. The study sample also reported to have perceived difficulties in emotion perception during the pandemic. Using the three subscales of the Toronto Alexithymia Scale (TAS-20), the participants were instructed to rate whether they experience difficulties in emotion perception relative to before the pandemic situation. Especially difficulties in identifying and describing feelings were reported. Moreover, the sum scores of the TAS-20 were significantly correlated with the students’ anxiety scores and the intensity of self-reported depressive symptoms (see Table 2). Taken together, these results are of particular interest in light of discussions which mental health interventions might help university students to cope with the threat provoked by the pandemic situation. Given that previous research has shown that high scores on the TAS-20 promote psychopathology [28, 29], the reports of the students about them perceiving difficulties in identifying one’s feelings in response to the pandemic situation relative to before the pandemic outbreak should be taken seriously and investigated in further studies in larger student cohorts.

### Worries about health and health behaviour during the COVID-19 pandemic

Moreover, the university students’ worries about health should be taken seriously. Chronic worrying is a sign of chronic distress and constitutes a risk factor of later development of general anxiety disorder [54]. In the current study, 65.5% of the final student sample (n = 220) reported being worried about their mental health and 71.4% reported to worry about their physical health more often than before the pandemic. The majority of the student sample did, however, not report to pay more attention to bodily sensations or symptoms (taste, smell, cardiovascular, respiration/breathing) than before the pandemic. However, worries about mental and physical health were accompanied by perceived changes in health behaviour. The percentage of “yes” and “no”-answers differed significantly for changes in health behaviour related to eating and physical activity behaviour since the outbreak of the pandemic. We did not ask the students for their eating behaviour or their physical activity level before the pandemic. Thus, the questions asking for perceived changes during relative to before the pandemic might have the potential of a memory bias. Nevertheless, pre-pandemic surveys report that up to 30% of university students do not exercise at a regular basis and do not meet the WHO’s weekly or daily physical activity recommendations (for an overview see [55]). The present results suggest a reduction in physical activity during the pandemic and physical inactivity and sedentarism are among the major risk factors promoting negative lifestyle-related diseases in the long run [55].

### Learning behaviour during the COVID-19 pandemic

The pandemic might have negative effects on student’s teaching and learning behaviour. In the present sample of university students, difficulties in teaching and learning were reported by the majority of students. One interpretation of these results is, that pandemic situations such as the current COVID-19 pandemic are characterized by uncertainty, fear, and threat, i.e., factors that are known to impact self-regulation. Previous research has shown that self-regulation is negatively related with threat perception [27] because responding to fear, anxiety and to threatening events depletes top-down control and self-regulatory resources [56, 57] that are also required for academic performance. In line with this, students reported having difficulties in focusing and concentrating on the teaching content during the current COVID-19 pandemic situation (see Fig. 4b). Self-learning formats such as e-learning may accentuate these effects.

### Self-concept and personality of university students, and machine learning

When asked to describe themselves with a modified version of the TST asking for descriptions of the students’ “actual self”, positive word use outweighed negative word use. When the student sample was considered as a whole, linguistic analysis of word use (see Fig. 5) supported a clear bias towards positivity that also accords with previous results that seeing yourself in a positive light correlates with positive self-descriptions and preferential processing of positive words [58–61]. Although this result must be seen in relation to a general positivity bias in written and spoken language (most languages having more positive than negative words [62], the analysis of word use suggests that the pandemic situation at the time of the survey did not provoke a threat to the self-concept of this university student sample and this, although linguistic analysis of the answers to the prompt that asked for feelings during the pandemic (see also Fig. 5) revealed a negativity bias as immediate negative responses to the pandemic situation in line with the results observed for the survey items asking for threat perception. Symptoms of state anxiety and current depressive symptoms may therefore reflect temporary changes of the university students to the pandemic situation that however occur immediately in response to the pandemic lockdown.

Psychological theories agree that individual factors such as one’s personality are correlated with subjective experience, well-being, mental health, and behaviour, e.g., [63, 64]. In line with this, analyses showed correlations between the Big Five (BFI-40) personality traits and the university students’ self-reported symptoms of anxiety, depression and their perceived difficulties in emotion perception. Statistically, correlation analysis, linear regression analysis, multivariate structural equation models, mediator analysis, or moderator analysis may all be feasible statistical methods to describe the relationship between psychological variables. However, in the present study we attempted to apply supervised machine learning algorithms that are built on regression models to further explore whether personality traits were not only correlated with mental health variables but could be predicted from the self-reported subjective experience of the participants obtained from this survey’s multimethod assessment. The observed results are promising despite the relatively small datasets used for training and prediction. The algorithms provided relatively accurate models for the prediction of personality traits from self-report data. As illustrated in Table 3, neuroticism as one of the big five personality traits (shown to be related to mental ill health [63, 64]) and in the present study sample significantly correlated with both, self-reported anxiety and depressive symptoms (see Table 2) could best be predicted by changes in current anxiety (threat perception, difference scores state vs trait anxiety), by the students’ self-reported trait and state anxiety, by their self-reported perceived difficulties in emotion perception (describing one’s feelings reported on the TAS-20), by self-reported changes in physical health behaviour (eating) and by self-reported difficulties in social distancing. Very recent results from surveys investigating the role of personality factors during the current COVID-19 pandemic also found that people’s self-reported psychological perceptions of and reactions towards the pandemic also depend on stable personality traits including the Big Five (for an overview [65]). Interestingly, there is also evidence that expression on personality traits such as the Big Five can change in conjunction with mental ill health [66]. Our results and these recent results suggest that future studies exploring the psychological consequences of the COVID-19 pandemic should include the assessment of personality traits in their anamnestic exploration of mental health and self-reported experience.

## Limitations

The present study adds to the evidence reported in the literature about the negative consequences of the current COVID-19 pandemic on mental health and well-being of university students. By using a mix of self-report measures it allows detailed insight into the subjective experiences associated with the pandemic in this population group in the psychological domains of mental health, health behaviour change and learning. However, some limitations already discussed in the sections above should be stressed. First, there was a high drop-out whose percentage was within the upper range of the expected drop-out rates for online surveys (20–50%). Although drop-outs were statistically assessed and compared to the final sample as far as appropriate, suggesting no bias by age or gender or the student’s personality, the drop-out reduced the final sample size reducing the power of the study. Thus, further data is required to demonstrate the generalizability of the present observations and to further explore possible cultural differences. In the present study sample, the reported significant differences between gender and students studying in Egypt or Germany might be tentative due to the small study samples. Power calculations suggest an ideal sample size of about N = 271 (90% confidence) or N = 385 (95% confidence) participants (margin of error of 5%). Although this sample size was reached in the beginning, it was reduced by the successive drop-out across the blocks of survey items. Second, statistics revealed significant results for the quantitative measures, however, the results of the linguistic tasks (self-concept and feeling prompts) could be reported only descriptively. The LIWC software was used for linguistic analysis. This allowed word categorization with high accuracy and validity [46] providing interesting insight that otherwise might have gone unnoticed and confirmed the results obtained from quantitative measures. Third, due to the small sample size the machine learning approach is exploratory and challenged by limitations. While machine learning tools have already been applied in many domains of psychology (e.g., in the domain of Affective Computing and Health Psychology), their use is still relatively under investigated in studies using psychology data obtained from multimethod approaches as the current one [67]. Existing studies using machine learning for analyzing personality- and behaviour-related data, mainly target personality prediction from larger datasets (e.g., [68]). In the present study, we followed guidelines and recommendations from existing machine learning studies discussing possible solutions for application of machine learning tools with small sample sizes (see for an overview [69–71]), using sample size of about 200 and support vector machines (SVM similar to SVR used in our study) for estimation of depressive symptoms, for personality trait and perceived stress prediction based on sample sizes ranging from 150 to 250 participants [69–71], as in the present study. In line with these previous studies applying machine learning tools to smaller sample sizes, we applied machine learning to a mix of measures that captured subjective experience in relation to the current COVID-19 pandemic situation in line with the recommendations from psychologically-driven computational approaches that suggest to include trait and state measures for prediction [25, 26]. Nevertheless, the present approach is exploratory and application of machine learning to small sample sizes need to be critically discussed, e.g., for a detailed discussion see [48], as it can lead to overfitting or overestimation. One recommendation to avoid such problems with small sample sizes is to use nested cross validation and control feature-to-sample ratio [48]. It will be interesting to follow-up the present ML results in future COVID-19 survey studies and use additional data collected during the course of the pandemic for validation and training in order to confirm the results from ML in hopefully larger samples, supporting the combination of machine learning and classical data analytics in the domain of psychology.

## Conclusion

This survey investigated the subjective experience of university students studying in Egypt or Germany during the COVID-19 pandemic in May 2020, i.e., in the time period after the first pandemic lockdown in the countries. Perceived changes in all psychological domains including state anxiety, depressive symptoms, threat perception, emotion perception, worries about health and behaviour (health, social distancing, and learning) were reported in the majority of students taking part in the survey. Recent COVID-10 surveys report similar high prevalence rates among university students across the globe [3, 4, 12, 13]. Although the results of this survey are tentative, the multimethod approach of this survey, using multiple scales, descriptive, correlational, and linguistic analysis, provides a valuable contribution to previously published COVID-19 studies. Moreover, the approach of combining descriptive analysis with machine learning should and could be followed-up in larger samples during the second period of the current pandemic. Crucially, despite the small sample size, the present results of self-reported anxiety and depressive symptoms among university students, that also seem to be supported by recent surveys including university students from other countries [3, 4, 12, 13] should be taken serious as they suggest that there is an urgent need to develop interventions that help prevent mental health among university students in order to avoid negative consequences in health and learning behaviour in response to the pandemic and provide health care to those students who might be at special risk of mental ill health.

### Questionnaire/survey

The questionnaires and self-assessment scales used in this study are standardized questionnaires and standardized scales whose references are cited in the manuscript in brackets. The single survey questions e.g., health and teaching have been developed for the purpose of this survey and are summarized in Table 1 in the manuscript. An overview of the online survey can be found in the supplement of this manuscript.

## Supplementary Information

**Additional file 1**. An overview of the online survey items and questionnaires.

## Data Availability

The datasets used and/or analysed during the current study are available from the corresponding author on reasonable request. Due to the informed consent form in which the possibility of raw data being published online was not explicitly stated, the raw data cannot be made accessible in online repositories.

## Abbreviations

BFI-40: BFI five inventory [43]
DTR: Decision tree regression
GBR: Gradient Boosting Regression
LIWC: Linguistic inquiry of word count [46]
PHQ-2: Personal Health Questionnaire 2 [38]
RMSE: Root mean squared error
SAM: Self-Assessment Manikin scales [41]
STAI: Spielberger Trait State Anxiety Inventory [37]
SVR: Support Vector Regression
TAS-20: Toronto Alexithymia Scale [42]
WHO: World Health Organization
