# Supplementary material for: How do you feel during the COVID-19 pandemic? A survey using psychological and linguistic self-report measures, and machine learning to investigate mental health, subjective experience, personality, and behaviour during the COVID-19 pandemic among university students
Source: BMC Psychol. 2021 Jun 2;9:90. doi: 10.1186/s40359-021-00574-x (PMC8170461; doi:10.1186/s40359-021-00574-x)
Supplement: Supplementary file 1 — Additional file 1. An overview of the online survey items and questionnaires. [file 40359_2021_574_MOESM1_ESM.docx]

**Detailed description of the online survey: How do you feel during the COVID-19 pandemic?**

**Psychological Domains, Questionnaires and Survey Items in chronological order**

| **Sociodemographics**  **General Information:**  **At which university do you study?**  Please write your answer here:  **_____________________________** |
| --- |
| **Demographic Data:**  **How old are you?**  Please write your answer here:  **_____________________________**  **What gender are you?**  Female  Male  Prefer not to say  Other: _____________________  **What is your country of residence?**  Please write your answer here:  **_____________________________** |
| **Personality**  Personality - The Big Five Inventory (BFI-40 [43])  Here are a number of characteristics that may or may not apply to you. For example, do you agree that you are someone who likes to spend time with others? Please write a number next to each statement to indicate the extent to which you agree or disagree with that statement.  **I see myself as someone who …**   - **BFI-40 items belonging to the subscales of the BFI-40 [43]:**   - Neuroticism, - Extraversion, - Openness, - Conscientiousness, - Agreeableness  **Anxiety**  Stait-Trait Anxiety - The Spielberger State-Trait Anxiety Questionnaire [37]  **How do you feel right now, in light of the Coronavirus? Please choose the appropriate response for each item:**   - **STAI items related to the STAI-State subscale [37]**   How do you generally feel (regardless of the pandemic)? Please choose the appropriate response for each item:   - **STAI items related to the STAI-Trait subscale [37]** |
| **Study Behavior**  Teaching and Learning (survey items created for this survey study)  **Considering the current situation during the Corona pandemic, please answer the following. Please choose all that apply:**  I cannot focus  I am preoccupied by the current situation  Other: ___________________________________ |
| **Current Situation**  Threat Perception (survey items created for this survey study)  The questions in this section always refer to the current situation imposed by the Coronavirus.   1. **How does the current situation make you feel? Please choose all that apply:**   Neutral  Happy  Sad  Angry  Surprised  Disgusted  Afraid |
| **Current Situation**  Threat Perception (survey items created for this survey study)  **2. How positive/negative is your emotion toward the current situation?**  - Answers on 9-point Self-Assessment Manikin (SAM) scales [41]: valence (1=negative, 9=positive)  **3. How strong is your emotion toward the current situation?**  - Answers on 9-point SAM scales [41]: perceived arousal of the current emotional  response, 1=low, 9=high  **4.** **How much control does your emotion toward the current situation have over you?**  - Answers on 9-point SAM scales [41]: perception of dominance/control of current emotional response (1=low, 9=high) |
| **Self-Concept**  Linguistic Task (shortened and modified for this survey study according to Twenty Statement Test [45]):  **WHO AM I**  **Write down five different answers to the question “Who am I”?** Don’t worry about the logic or the importance of your responses – just write down the answers as they occur to you.  I am ____________  I am ____________  I am ____________  I am ____________  I am ____________ |
| **How do you feel during the current pandemic?**  Linguistic Task (created for this survey study):  **How do you feel during the current pandemic?** You can complete the statements with the phrase “I am/feel” and add a verb, noun or adjective.  I am/feel ____________  I am/feel ____________  I am/feel ____________  I am/feel ____________  I am/feel ____________  **Health behaviour and worries about health**  (survey items created for this survey study)  **Please answer the following questions**:  I think I have put on weight.  Yes No  I think I have lost weight.  Yes No  I eat more than before the pandemic.  Yes No  I eat less than before the pandemic  Yes No  I sleep more than before the pandemic  Yes No  I sleep less than before the pandemic  Yes No  I am exercising more than before the pandemic  Yes No  I am exercising less than before the pandemic  Yes No  I am worried about my mental health more than before the pandemic  Yes No  I am worried about my physical health more than before the pandemic  Yes No  I am worried about my weight more than before the pandemic  Yes No  I am worried about my weight less than before the pandemic  Yes No |
| **Bodily Symptoms**  (survey items created for this survey study)  **Please answer the following questions.**  **Changes during the pandemic …**   \| **1** \| **2** \| **3** \| **4** \| **5** \| **6** \| **7** \| **8** \| **9** \| **10** \| \| --- \| --- \| --- \| --- \| --- \| --- \| --- \| --- \| --- \| --- \|   **not at all /decreased no change very much/increased**  …. smell  …. taste  …. listen to your heart (heart beats)  …. listen to your breath (breathing)  …. listen to your body (symptoms)  …. appetite/eating/drinking  **Social Behavior**  (survey item created for this survey study)  **Difficulties in not going out during the pandemic?**  Yes No |
| **Difficulties in Emotion Perception**  Toronto Alexithymia Scale (TAS-20 [42])  **Do you think your communication, thinking, and feelings have changed during the pandemic?**   - **TAS-20 items belonging to the three TAS-20 [42] subscales:** - difficulties in describing feelings, - difficulties in identifying feelings, - externally oriented thinking |
| **Depression**  Screening (Patient Health Questionnaire – PHQ-2 [38]):  **How much do you feel affected by the current pandemic?** Over the last two weeks, how often have you been bothered by the following problems. Please chose the appropriate response for each item:   - **PHQ-2 items [38]** |
